# Learning from the implementation of clinical empathy training: an explorative qualitative study in search of the barriers and facilitators

**DOI:** 10.1186/s12909-022-03877-8

**Published:** 2022-11-23

**Authors:** Luca C. Barak, Giliam Kuijpers, Lotte Hoeijmakers, Fedde Scheele

**Affiliations:** 1 OLVG Hospital, Amsterdam, the Netherlands; 2 Social enterprise MedGezel, and OLVG hospital, Amsterdam, the Netherlands; 3 Athena Institute, VU University Amsterdam, and Amsterdam UMC, Amsterdam, the Netherlands

**Keywords:** Empathy training, Empathy skills, Staff culture

## Abstract

**Background:**

Amid concerns about the decline of empathy during the clinical training of medical clerks, evidence that empathy improves patient outcomes suggests some potential for teaching empathy in ways that will affect the knowledge, attitude and behaviour of medical clerks. This potential alone cannot, however, guarantee the success of educational innovations to introduce empathy to the medical curriculum. This research aims to identify the barriers and facilitators of the implementation of a specific clinical initiative to enhance the empathy skills of clerks, namely the training of clerks to act as a ‘MedGezel’ or ‘medical coach’.

**Method:**

We conducted an explorative qualitative study based on interview data collected and analyzed using reflexive thematic analysis and the readiness for change theory. We conducted semi-structured interviews with relevant stakeholders in this particular qualitative study. Thematic analysis was based on open and axial coding using ATLAS.ti 9, which facilitated the emergence of common themes of interest and meaning for the study.

**Results:**

A total of 13 relevant stakeholders participated as interviewees in our study. The data was collected from April to June 2021. Our analysis generated 6 main themes which can provide insights into why the implementation of the MedGezel educational innovation failed so far. The following themes emerged: the case for change: why change?; practical necessity; leadership; management and resources; staff culture; and alignment with the corporate strategy.

**Discussion:**

The implementation failure can be partially explained as resulting from the personal attitudes and choices of participants, who struggled to reconcile a vision that they liked with side effects that they feared. While participants repeatedly mentioned management and leadership issues, these organizational issues seemed less important as they could be easily resolved in practice. What was more important and fatal for the initiative was its lack of alignment with staff culture, despite its alignment with corporate strategy.

**Conclusion:**

This investigation into the barriers and facilitators influencing the implementation of the MedGezel program identified 6 explanatory themes, the most impactful one being staff culture.

## Background

Humanism in care is characterized by interest in and respect for the patient’s concerns and values regarding psychological, social and spiritual domains. The time and energy spent on teaching this attitude has to be balanced with the time and effort spent on teaching medical expertise [1]. There is evidence that patient outcomes are improved by empathy, a hallmark of humanistic care [2–4], and that the teaching of empathy has an effect on the knowledge, attitude and behaviour of medical clerks, i.e. medical students in their final and clinical years of medical training [5]. This research focusses on the factors that influence the implementation of a practical initiative to enhance the empathy skills of medical clerks.

Medical clerks are educated in teaching hospitals, where they learn various clinical skills. They work under the supervision of physicians. There are concerns about the decline of empathy during the clinical training of medical clerks [3]. The literature suggests that a significant decline in empathy occurs during the third year of medical school. The erosion of empathy occurs during a time when the curriculum is shifting toward patient care activities, when empathic behavior is most essential [3]. There are ongoing debates about the definition of empathy, which is a poorly understood concept that is often confused with other terms, such as sympathy and identification. Empirical research on empathy among medical students has been hampered not only by conceptional confusion but also by a lack of broadly accepted instruments to measure empathy in the context of medical education [5]. Although some literature suggests that educational interventions can be effective in maintaining and enhancing empathy in undergraduate medical students, there is still much debate about the different strategies due to the lack of conceptual clarity regarding empathy and the lack of knowledge about the long-term effectiveness of the interventions [6].

Even though there is a lot of debate in the literature about empathy-based education, we made a choice from a practical standpoint to implement a grass root initiative. This initiative was designed to enhance empathic behavior by teaching medical clerks compassionate interactions, deep listening and paying personal attention to each patient’s life (see Table 1). During the implementation of this empirical empathy-based intervention, we encountered several difficulties and obstacles, which prompted us to investigate the possible practical reasons why the implementation failed.

**Table 1:** MedGezel

In the last few years, we created and tested a prototype of an educational intervention to teach medical clerks empathic behaviours by acting as a medical coach (a MedGezel), guiding a patient in obtaining a patient-centered doctor’s consultation. Our educational methodology is based on making medical clerks more aware of human relations. The program is made in a way to create learning conditions and an encouraging context to facilitate the innate empathic abilities of the individual clerks rather than trying to teach directly “how to be empathic”. We work from the idea that empathic behavior is consequence of a resignification of human relation without a predetermined format and that it’s not possible to create a mold from where medical clerks can be molded to become more “empathetic”. Empathic behaviour is a humanistic trait that each of us possesses to some degree. Which can be stimulated. Our educational and practical method can be turned into a lifelong practice where (future) doctors are engaged in real human connection. The clerks learn to find their authentic voice to communicate from their intuition and not in a “zombie”-like manner [7]. This new educational empathic health concept already won several national and international awards and nominations because of its promising value for future person-centered healthcare. Because we started very small and outside an official research setting, we cannot provide the exact numbers, but the following approximate numbers are very close to the real data: more than 100 medical students selected from all the medical faculties in the Netherlands underwent a voluntarily training as medical coaches, and they guided approximately 100 patients during their care trajectory in several Dutch hospitals. Special emphasis was placed on compassionate interactions with the patients via deep listening and personal attention to the lives of the patients. A coach empowers the intrinsic ability of people to take charge of their own wishes, needs and challenges [8], based on the understanding that people find their own answers to their personal challenges. To become a coach for others, one has to first look at one’s own beliefs, wishes and challenges [9]. Truly empathic behaviour is authentic and comes from a place of deep knowledge of oneself and the other. By stimulating awareness of one’s personal feelings and thoughts in relation to the other and practice an unfolding coaching conversation we shift the focus of external imposed behaviour to behavior directed by felt sense. As such, our medical coach training consists of two crucial components: getting to know oneself and getting to know the patient. After the medical coach training, the MedGezel prototype of empowering patients consists of three stages: 1: before the consultation with the doctor, the medical students connect with the patients to discuss their personal wishes, needs and challenges. The medical students prepare the patients for the consultation by rehearsing the most important questions. 2: During the consultation with the medical specialist, the medical student acts as a patient advocate and buddy of the patient. 3: After the consultation, the medical student repeats the most prominent features of the conversation with the medical specialist and makes sure that everything is well understood by the patient. The medical student also makes sure that the feelings and concerns of the patient are met with dignity and respect. As collateral gains of deploying a MedGezel, patients may feel better understood and better prepared for their appointments, which gives physicians more opportunities for patient-centered care.	

As we attempt to implement empathy in the formal undergraduate curriculum, it is important to understand the actual clinical practice as well. The medical world is characterized by a powerful and intensive socialization process that has sometimes been referred to as the “hidden curriculum” [1]. This socialization process is revealed in informal forms of control over group functioning, as inter-personal behaviour and relations of authority are structured by unwritten rules [10]. This hidden curriculum is highly influential and may easily overrule the knowledge gained and the attitudes achieved in previous course work. Furthermore, it may shape the future attitudes and behaviours of a clerk [11]. It is through this hidden curriculum that a specific professional culture is transferred and maintained, and since it is evident at so many different times and in so many different places, it may well be referred to as a common professional identity, or the medical “habitus” [10, 12]. In the practical initiative under study in this manuscript, attention for empathic health care may require a more prominent position of empathy within the medical habitus.

Branch et al. [1] provided practical tips on how to establish a climate of humanism. These tips include recognizing and using learning opportunities, using role modelling, making sure that the learners are actively engaged, being practical and relevant, and using multiple up-to-date strategies at the same time. From the perspective of change management, the approach of Branch et al. is attractive because it does not demand a structural change in the clinical workflow and the alignment is relatively good [13]. In our opinion, a sensible further step to take is to optimally engage clerks as part of the health care team [14] by making them stewards of humanistic care. They need to become practicing, engaged and responsible team members [14], they need to reflect on their own actions and on the humanistic care climate that they are part of [15], and finally, they need to rehearse their roles [5]. Based on these principles, we decided to train clerks as medical coaches and to prepare them for a responsible role as stewards of humanistic care. Our focus for the learning effect was on empathy and person-centered care (see Table 1). For the pilot, a coordinator was assigned to assist in explaining and planning the initiative at the outpatient clinics.

At the start, the pilot was met with enthusiasm from multiple academic and affiliated training hospitals, we won a national health innovation prize (2020), and national media reported positively about the project. However, when the time came to make concrete arrangements with hospital departments on how to implement this innovation, we hit “a wall of goodwill”. There is much goodwill in relation to the vision and the idea of training the medical clerks to become medical coaches. Furthermore, this practical innovation of the medical curriculum matches official policies concerning patient participation. Nevertheless, the “wall” that we encountered was characterized by hesitancy to actually move forward with the proposed changes. We hypothesized that despite all the enthusiasm and goodwill, the stakeholders, the organization and/or the medical culture were not ready for this kind of change.

In this current period of transition in health care [16] as well as in the training pedagogy that tries to prepare for it [17], it is important to understand the problems that arise when challenging educational innovations are implemented in the medical curriculum. This is especially the case when these innovations are not entirely aligned with current practice [13] and even ask for a change in the medical habitus [12]. As such, it is necessary to understand why change programs become implemented or not. According to the theory of readiness for organizational change, an innovation needs to satisfy a specific need and add value to the context by which it needs to be adopted and routinized into standard practice [18]. To create readiness, a sense of urgency needs to be established, team members need to be empowered, and an appealing vision must be offered along with the confidence that it can be realized. The implementation progress is accelerated in the presence of good leaders who are fully equipped with toolboxes of change management principles. Change is seen as a social process that requires creativity, a sense of ownership and the support of multiple layers of an organization [19]. The concept of readiness for change may assist us in understanding the nature of the “wall of goodwill” in our case and in identifying its most important sources. Literature on innovation failure in medical education practice is scarce [20], and contributing to the literature what can be learned from implementation successes and failures may add to our understanding of change management principles in the context of clinical learning processes. Learning from mistakes is a powerful instrument for advancement [21].

In the present study, we aimed to answer the following research question: which clarifications result from a change perspective on the obstacles encountered during the implementation of a practical empathy training for clerks in patient care? A better understanding of the “wall of goodwill” against curriculum changes for empathy training is especially important in view of the ongoing transition in healthcare towards patient-centered care.

## Methods

### Setting

This study focused on identifying the factors that influence the “wall of goodwill” against the implementation of an empathy educational innovation. We conducted the study in the Netherlands, where the pilot of MedGezel took place in training hospitals.

### Study design

Since empathy training is an under-researched domain, we conducted an explorative qualitative interview study using the reflexive thematic analysis (RTA). This analysis is a flexible interpretative approach to identify and analyze patterns or themes in a qualitative data set. RTA is about “the researcher’s reflective and thoughtful engagement with their data and their reflexive and thoughtful engagement with the analytic process” [22]. In this study, we used the knowledge of four coders in a beneficial reflexive manner to sense-check ideas and explore multiple assumptions and interpretations of the data. The aim was not to provide the ‘correct’ answer, but to achieve richer interpretations of meaning in a collaborative and reflexive way [23]. In this way, we were able to gain a deeper insight into the subjective thoughts of the participants about the possible barriers and facilitators of implementing the proposed innovation. Moreover, the influence of the researchers’ interpretations of the research data is taken into account.

### Semi-structured interviews

Semi-structured interviews were conducted with the relevant stakeholders to collect rich data. A semi-structured approach was chosen because it allows for more detailed responses and additional questions. It invites the interviewee to talk more about their own attitudes, beliefs, behaviors or experiences [24]. To gain more insight into the readiness for change themes in medical education, we used topics derived from the specialty training’s organizational readiness for curriculum change (STORC) questionnaire. STORC was validated to diagnose possible hurdles in implementation processes and to perform specifically targeted interventions when needed [25]. An interview agenda was composed to ensure that certain main topics were addressed in the interview. The participants were interviewed about their thoughts regarding the implementation of patient-centered care, their perception of empathy in the consultation room and their ideas concerning the implementation of the medical coach model.

### Participants and sampling procedure

To gain rich data and to widen the scope of this study, the perspectives of the following four kinds of participants were sought: 1) physicians who participated in the MedGezel pilot as well as physicians who did not participate (further named Physician); 2) educators who are responsible for education in their respective departments (further named Educator); 3) managers involved in the educational infrastructure for medical clerks in clinical practice and marketers (further named Quality department); and 4) medical clerks in the rotation phase of their training (further named Medical clerk). Recruitment of participants continued until theoretical data saturation was reached.

### Data collection

All semi-structured interviews were conducted by the main researcher (LB) online via ZOOM or in private offices between April 2021 and June 2021. Selected potential participants were contacted to inform them of the nature of the research, the aim of the study and the value of participation. An appointment was made, and they were asked to sign a letter of consent before the interview. The interviews were audio-recorded, transcribed verbatim and reviewed for accuracy by the main researcher (LB). The audiotapes and transcriptions were anonymized for everyone except the researcher and stored in a specifically designed safe environment for research data.

### Data analysis

In the reflexive thematic analysis approach, the process of coding and theme development will evolve throughout the analytical process as shown in Table 2. By facilitating familiarity with the data, interpretations of new patterns will produce themes derived from the data, around a core organizing concept, in our case the readiness for change theory. We started the “familiarization” phase with listening to and re-reading the entire dataset in order to become intimately familiar with the data and to identify appropriate information that may be relevant to the research question. After that, the process of open and axial coding was undertaken to produce descriptive labels which summarized pieces of information from the transcripts. For the open and axial coding, we used ATLAS.ti 9, a qualitative data analyzing software program. After all the relevant data items had been initially coded, a process of continuous comparison and interpretation aggregated layers of meaning across the dataset. We then reviewed and combined the initial codes according to shared meanings between the researchers so that they formed initial themes. During the last phase, based on the detected themes and informed by the organizational readiness for change theory, the initial themes were reviewed during discussions within the author team. During this process, which was alternatively inductive (collecting interview data) and deductive (categorizing, detecting themes), the level of abstraction increased and apparent relations between over-arching themes became visible. In this inductive and deductive analysis, the recurring commonalities between the different items from the data set were identified regarding the organizational readiness for change theory. After the first coding and categorization rounds, a second researcher addressed possible differences in the coding and categorization process until major consensus between the researchers was reached. Using the organizational readiness for change theory helped to make the emerging themes more generalizable and increased confidence in their validity [24].

**Table 2:** Reflexive thematic analysis: the six-phase analysis (adapted from Braun and Clarke (2006)) [26]

Analytic phase	Description
Data familiarization	Becoming intimately familiar with the data by transcribing and re-listening to the audiotapes to be able to identify patterns
Initial code generation	Producing descriptive labels which summarize pieces of information from the transcripts that may be relevant for the research question
Generating (initial) themes	Comparing and interpreting labels to produce initial themes and identifying meaning and relationships between these initial codes
Theme review	Reviewing initial themes informed by the readiness for change theory
Theme defining and naming	Formulating final themes by defining over-arching themes in order to respond to the research question
Report production	Writing a compelling story told by the data, within and beyond the simple descriptions of the themes

### Reflexivity

Choices about identifying and selecting more complex categories and themes based on the reflexive thematic analysis approach was done by four coders. Firstly, the main researcher, BSc and PhD candidate Luca Barak, who is a young researcher in this field, with an interest in compassion-based learning and how to implement that learning in medical education. Secondly, the co-researcher, physician, social entrepreneur, curriculum designer and trainer of medical coaches Giliam Kuijpers, who as a physician herself became a patient and noticed that empathic behavior between physicians and patients could be improved. She became an expert in social innovation and designed and developed the training for the medical coaches according to her own experiences. Thirdly, the second researcher, who helped in coding and categorizing, physician and manager of education, Lotte Hoeijmakers, who encountered difficulties guiding and supporting the medical students under her supervision. Finally, Fedde Scheele, who complemented our team as a professor and expert in change theory, with a long scientific carrier in health innovations and change management. Though the primary background of the four researchers is the medical profession, nowadays they work in different fields, such as education, change management, social innovation and project management. Therefore, the coding of the data took place from different perspectives. We regard the development of the training and the implementation of the medical coaches concept as a grassroot initiative. This practical initiative was developed based on clinical experiences and was not designed for scientific reasons. Keeping this in mind, we used the concept of readiness for change as a useful theoretical framework.

## Results

A total of 23 relevant stakeholders were invited to participate in our study, 13 of whom agreed. Of the included participants, 6 were specialists, 2 were clerks, 3 were educators and 2 were involved in quality departments. Of the specialists, 4 had a double function, as a physician as well as working in either the quality department or in undergraduate education. All the participants held positions in one of four training hospitals in the Amsterdam area: the OLVG hospital, Spaarne Gasthuis, the VUmc or the AMC. All interviews were conducted in Dutch, the language in which all the participants were highly proficient. The interviews lasted between 30 and 90 minutes each and were held in a period of 3 months, from April to June 2021. After conducting 13 interviews, data saturation was reached, so there was no need to invite new participants.

Our analyses resulted in six main themes found within the concept of readiness for change. These themes can provide insights into why the implementation of the MedGezel educational innovation failed. The themes that emerged were described as follows: the case for change: why change?; practical necessity; leadership; management and resources; staff culture; and alignment with the corporate strategy.

### The case for change: why change?

The case for change in the form of MedGezel revolved around the need for a structured way of bringing more empathy and a better perspective on the patient journey to the training of future doctors. During our interviews, we tried to discover participant perceptions regarding the MedGezel proposition. Our results indicated that most of the participants perceived the proposition of a change towards a new empathic program as being a necessary innovation. They stated that the innovation provides several advantages, such as the clerk learning to see the patient’s viewpoint, the patients feeling more seen and heard, and the specialist knowing what matters the most for the patient. The following quotes provide evidence for this interpretation:

*“We saw the needs on both sides; with the patients, but also that it’s good in the training for clerks, because we also know that there are clerks who find it quite difficult to think from the patient’s point of view.” (Physician)*

*“I strongly believe that it has added value for the patients, because there is someone sitting next to them who also has a bit of medical knowledge and who prepares the consultation with the patient.” (Educator)*

*“It is always nice for the specialist if there is a clear question, because otherwise we will have to fill it in, and that might not always be correct.” (Physician)*

The first impression, therefore, was that the proposed innovation elicited general goodwill, although some participants expressed second thoughts. A minority of the participants argued against the necessity of change towards more empathy in the medical education. These participants indicated that the innovation was rendered unnecessary by other ongoing similar initiatives to make doctors more patient-oriented: *“We are already listening very well and making decisions together, so why should we also use a MedGezel or why should we do a training “deciding together”?” (Quality department).*

Another participant said that sometimes empathy is not the main goal of the doctor’s consultation. This is especially the case with consultations with new patients, in which the main goal is to find out as much as possible about the medical problems, and at those moments little time is available for an empathic conversation. *“At some point, we have half an hour for taking the patient’s medical history, doing the physical examination and that’s it. Empathy is not the purpose of that consultation.” (Physician).*

Some participants argued that the decline of empathy is inevitable and that it is something that has to be accepted. They said that this might be due to the more difficult medical tasks a clerk or a freshly graduated doctor has to perform. Also, getting used to the difficult situations, such as seeing a lot of patients over time with the same terrible conditions, might make the experience less special and reduce the empathy of a doctor. One physician made the comparison to newly licensed drivers: *“You forget that a patient is vulnerable. When you are a young doctor, you’re much more concerned with switching, coupling in first gear, second gear, indicating directions. That is very different from surveying a traffic situation, whilst that might be the most important for a patient.” (Educator).*

An educator said that strong competition within educational departments creates a fear of losing hard-fought time on the program whenever an innovation is introduced. Therefore, they might not regard empathy education as a priority. *“That is the thing with patient perspective educational programs, they meander against the borders of medical ethics, medical psychology and also the educational curriculum of professional conduct. They all have to fight with every curriculum innovation to defend their hours in the curriculum training.” (Educator).*

One of the specialists questioned if empathy is even teachable, because feeling empathic is not the same as showing empathy. *“You can’t teach empathy, it’s in you. This means that some people are more and others less suitable for the profession.” (Physician).*

To summarize, among the participants, most had a positive attitude towards this innovation in the form of MedGezel. However, during the interviews a lot of reasons to question the necessity of the innovation arose. Moreover, some participants extenuated the fact that the innovation had not been implemented yet, due to not having the right conditions in order yet.

### Practical necessity

Most of the participants recognized the need for a medical coach to guide patients. They explained that the medical aspects of a patient can sometimes cloud a doctor’s view on the patient’s personal life:

“*The focus of the medical curriculum is on the medical aspects of a patient and the doctor’s view on a patient, it is less about the experiences of a patient and the world they live in.” (Physician)*

*“It is good for the clerk to see what happens when a patient walks out of the consultation room. What did the patient remember? What goes on in the patient? What kind of question will they have? We do not see that perspective.” (Physician)*

Most participants thought that for clerks to learn the patient’s perspective from the beginning of their training might have advantages, not only for their education but also for their career as a future doctor. One of the clerks mentioned: *“I will keep using some of the striking questions I learned, such as ‘What characterizes a good day and a bad one?’ and ‘What do you prefer to do in your normal day life?’.” (Medical Clerk).*

Furthermore, some of the participants mentioned that with a MedGezel, patients would have a sparring partner with whom they could reflect on their feelings and concerns: *“Some of the patients are very assertive and had their feet wetted before, others do need a sparring partner and are now able to reflect on their feelings with a clerk, who will learn from this as well.” (Physician).*

Although many participants mentioned the importance of a living coach who can interrupt and coach the patient, some questioned the necessity of the innovation regarding patient participation. In the educational department, one of the participants said that there are already multiple educational programs that are more patient-oriented: *“There are already other educational components, such as ‘the clerk follows a patient journey’.” (Educator).*

In their view, there were already certain digital questionnaires available which could help achieve more patient participation in the consultation room: *“At the moment, there is a new feature in EPIC (electronic patient file) that allows patients to send questions to the doctor in advance. With this feature, the patients are forced to think ahead more about their questions.” (Physician).*

In balancing the pros and cons, some argued that the time investment of the MedGezel innovation would be quite high, which raised the question of what added value it would contribute: *“You have to be very critical, how much work does it cost us and what does it get us?” (Physician).*

One educator expressed worries that the clerkship itself might be threatened by adding yet another activity, given that clerks already spend a lot of time on extracurricular activities. Moreover, some expressed concerns that some clerks might feel disadvantaged for missing out on more technical teaching moments while working as a MedGezel: *“The question remains: Will they miss something what the other clerks do get? They spent a lot of time on it, it was quite intensive.” (Physician).*

Altogether, the participants suggested that while the MedGezel innovation could expand a clerk’s deeper understanding of the person behind the patient, it could also be relatively too time-consuming, considering all the other priorities in the medical curriculum.

### Leadership

Leaders are often needed to carry an innovation forward, and some participants said that such leaders would truly have to believe in the innovation in order to get others to join them: *“In one of my projects, the program directors were involved, both people with a great heart for education, who have a vision on education, and if they are convinced that it makes sense, they will stand up for it.” (Educator).*

Also, one of the specialists with a quality assignment argued that sometimes it helped if the leader was someone who was respected and had built credibility in past projects: *“In the past, we worked well together, that is important, you know each other so you will not start from zero. You will think: Ok, fine, trustworthy.” (Educator).*

Most participants saw strong leadership as a possible facilitator to bring an innovation to the next level: *“What works is, I call it a trinity: a clinical champion, a head of department and a kind of change agent, which could be a very enthusiastic senior nurse, who stands on a soapbox announcing new ideas.” (Educator)*

### Management and resources

Some departments encountered minor difficulties with the implementation of MedGezel. Those departments agreed that the added value was more important and were willing to face the obstacles in a way of making this implementation work: *“At our department, the question ‘how do we solve this?’ was addressed by starting with only a few clerks to understand the possible technical difficulties, having two highly involved staff members and a strong feeling of importance for this kind of empathic innovations.” (Physician).*

Though for some departments the implementation encountered minor difficulties, most concerns mentioned by the other participants had to do with difficulties in the scheduling of patients and clerks:

*“To fit in a MedGezel, a new schedule needs to be made, and who is going to see the patients as a clerk when the clerk is working as a MedGezel at that point.” (Quality department)*

*“Who is going to select the patients? And who is going to call these patients?” (Quality department)*

An expert from the quality department stated that these mentioned difficulties could be overcome with a clear format: *“The schedule challenges could be solved with a format for the selection of the right patients and a new scheduling system within EPIC.” (Quality department)* Also, this expert sugested starting a project group with people who work as outpatient clinic assistants or secretaries in order to really understand what happens on the workfloor: *“It is very important to have a project group with people who really understand the work proces in the clinic.” (Quality department).*

Another concern was raised about financial contributions for the training of the medical coaches: *“Where will the money come from? Because somebody has to invest, the hospital or the department?” (Quality department).*

Even though the importance of empathy was clear to the majority of the participants, nearly all participants observed an organizational challenge in implementing the innovation: *“With the way the clerkship is organized now, we do not encounter a “not wanting”, but rather an organizational implementation challenge.” (Educator)*

### Staff culture

During the analysis of the results, we encountered two important but distinct perspectives on medical education. On the one hand, there was the perspective of the medical institution, which focusses on teaching complex clinical abilities to the clerks as well as on their role within group dynamics: *“Professional identity formation is a term in medical education. This is about how doctors develop themselves, not only in knowledge and skills, but also as a human being, because you want a doctor with good medical skills, who is also good in group dynamics and has social skills.” (Educator).*

The second perspective was that of the educational institution, whose concerns with assessment and accountability gave an important role to the measurability of education: *“Education is such a huge machine and it needs to be accredited and the language spoken is checklists and proving that it works. Effectiveness.” (Quality department).*

From the perspective of the medical institution, there are many concerns about the different roles a clerk has to fulfil in the event that they are also made to act as MedGezel. It was frequently mentioned that role confusion could arise among the clerks, which might hamper their education: *“The clerks might be hesitant to coach the patient and to ask in-depth questions in front of the doctor, because the clerk is the patient’s advocate in this setting, and the next moment his work will be evaluated by this same doctor.” (Physician).*

The other side of the coin of role confusion was also mentioned, i.e. that the physician might feel judged: *“I think that the physicians had the idea that they would be judged, which is kind of peculiar, because normaly a clerk will sit next to them and will have some kind of judgement about them as well.” (Physician).*

Even though clerks are not allowed to give medical advice to patients in their normal role as clerks, one specialist mentioned this concern: *“I have the impression that the clerk might go and sit on the doctor’s chair and answer all kinds of questions.” (Physician).*

One of the experts from the quality department said that the reason for this fear might be rooted in the conviction of older physicians that what they do is fine, given that they have been doctors for many years, and that this innovation represents a kind of criticism on their work: *“Some say: ‘I have been a doctor for 30 years, I’m a fine doctor’, though they do not listen that well and they are not willing to open up and learn to do better. It’s a kind of autonomy in saying ‘why? I’m doing well at my job’.” (Quality department).*

Besides this perspective, it was also mentioned that medical physicians have different views on the importance of educational innovations. One of the participants mentioned that doctors value science within the medical field more than innovations within the field of management or education: “*In practice you see that hard science and PhD trajectories are considered much more important than soft projects within education.” (Physician).*

From the perspective of the educational institution, there seems to be a feeling that an innovation needs to be initiated by the institution itself. One specialist hypothesized that the feeling of ownership needs to be deep-rooted in order to back up an innovation. This need is particularly evident whenever an innovation comes from an external source and is not ‘hospital-owned’. *“It’s not really from OUR hospital. There are, I don’t know how many FTEs of educators and doctors who develop education here, we can all do that ourselves and this all costs so much money that we are not going to do anything that comes from elsewhere.” (Educator).*

To summarize, there are concerns that the new role of the medical coach could disrupt the traditional connection between specialists and clerks as well as concerns about what this break could mean for the medical education department*.*

### Alignment with the corporate strategies

The analysis of the interviews revealed that an innovation can help to realize a corporate strategy. A marketer at the quality department explained that it becomes increasingly important for hospitals to distinguish themselves from other providers of healthcare in order to improve their position on the market: *“We use marketing to distinguish our hospital from other hospitals in the district.” (Quality department)* She explained that this branding could be supported by an innovation like MedGezel: *“Once you’ve defined your branding and your values, the innovation should fit that branding.” (Quality department).*

This view was shared by the educational department, with one educator mentioning that an innovation can be used to distinguish a university from others: *“At the moment of selecting a university hospital, one of the questions is: what characterizes your education?” (Educator)* She also said: *“I think we can use MedGezel as an interpretation for the core values of this university hospital.” (Educator)* An expert from a quality department mentioned that MedGezel fits in a learning organization and a people-orientated organization: *“This fits seamlessly in a learning organization and a people-orientated organization, in which ‘people’ can be defined in different ways: as a clerk, as a patient, but also as a medical specialist.” (Specialist)* The same participant also mentioned that many policy plans have already been made years ago to reach organizational goals that include training patients and clerks in patient participation, and MedGezel might be a good initiative to accomplish these goals: *“This innovation touches many organizational goals in teaching patient participation and training the patient. These themes have been in policy plans for years, but those memos are gathering dust on the shelf.” (Specialist).*

### Discussion

This study sheds light on the factors that influenced and hindered the implementation of the MedGezel clinical education initiative, which was intended to enhance the empathy skills of medical clerks by placing them in the role of medical coaches for patients.

Current systems of care and the biomedical paradigm create a risk of a decline in empathy in healthcare [27]. Both healthcare givers and patients may experience anxiety and stress due to the dehumanization of healthcare practices and processes [28]. To overcome this risk, a number of hospitals in the Netherlands chose to implement an empathy-based educational intervention. Despite initial praise for the initiative, the implementation was hindered by several unknown factors. We found that a majority of the stakeholders who participated in the present study embraced the vision of the initiative, but while some argued the need for empathy training, others felt that existing empathy training and performance were already sufficient. It became evident that leadership was essential for the success of any such initiative and should be a focal part of planning activities. In one department, management and resources were no obstacle for the implementation, while in others they were, despite the presence of a program coordinator. Participants expressed concerns about role confusion for clerks who not only had to play the role of a student, but also that of a patient’s coach and advocate. Despite these challenges, the general alignment of the initiative with corporate strategy was considered to be adequate.

Reporting on the factors that might have influenced implementation successes or failures is uncommon in medical education. Moreover, it is difficult to find other practical examples of similar educational change initiatives in the literature. However, reflection on findings of literature dealing with change management and professional culture provided useful insights into the “wall of goodwill” that was met by the MedGezel initiative.

At the personal level, we could recognize a struggle within the minds of the study participants. Most were keen to embrace the vision that inspired the MedGezel initiative because it fit their moral compasses. At the same time, however, many were concerned that changing routines would take some effort and were therefore tempted to maintain the status quo. We observed this same struggle during the implementation of shared decision making [29]. Moreover, literature suggests that health professionals overestimate the degree to which they have overcome their paternalistic attitude [30]. The outcome of this mental struggle may have prevented participants from taking leadership and acting as change agents for future health care [17].

At the organizational level, we knew that the MedGezel initiative was in alignment with the current hospital vision. In many countries, shared decision making by doctors and patients is recognized as being a part of daily learning [31], and an empathic quest for the patient’s preferences is considered appropriate for this process [32]. In addition, the international Institute of Healthcare Improvement (IHI) encourages and supports more meaningful conversations between the people who provide healthcare and the people who receive it [33]. Nevertheless, despite this strategic alignment, the initiative’s alignment with work floor routines was not perfect. Although the attending physician, the clerk and the patient were in the same consultation room as usual, the clerk would in certain patient contacts focus on person-oriented histories rather than on medical diagnoses. Focusing on these histories will constitute a disruption of the traditional connection between physicians and clerks, which might ask for small arrangements to make this change in connection somewhat easier. The evaluation after the consultation with the physician between clerks and patients may have disrupted initial the work of a medical clerk. Such changes in practice routines may seem small, but study participants attributed considerable importance to these changes. Routine changes are known to be difficult and time consuming in medical education [20].

The most important Achilles heel of implementing the MedGezel initiative may have been that it could be perceived as a threat to professional culture. By placing the clerks in the role of medical coaches and giving them the task of briefing and debriefing patients before and after a consultation with a physician, the MedGezel initiative risked upgrading the status of the clerk and making the physician an object of potential criticism, which would conflict with a well-studied subject in medical culture, i.e., ‘saving face’ [34]. Physician culture dictates that openly criticizing each other is to be avoided, whereas an open culture is needed to be able to discuss each other’s performance [35]. Studies of Witman [36] and Scheepers [37] in the Netherlands revealed flaws in the achievement of an open culture. Hierarchy issues also play a serious role and are part of our medical culture [38]. These issues became quite evident in narratives of hierarchy in hospitals, which revealed that trainees were expected not to challenge or question the judgements or actions of their seniors [39]. Habits, values, and attitudes, even dysfunctional ones, are seen as part of one’s identity, and for a clerk to potentially challenge a senior’s behaviour would be regarded as breaking unwritten rules [40].

Finally, we realise that the concept of empathy is a phenomenon that has been under discussion throughout history. According to the German phenomenologist Edith Stein, empathy cannot be forced to occur because empathy happens to us [41]. In alignment with this point of view, some researchers argued that the act of empathy is unteachable. On the other hand, it is possible to facilitate empathy, and conversely, empathy can be interrupted and blocked by an unfavourable learning context [42]. Future research should aim to gain a better understanding of the concept of empathy, the different underlying paradigms and their implications for designing future clinical empathy trainings.

### Strengths, limitations and future research

The strength of this study is its openness in reporting on and learn lessons from the barriers and facilitators of implementing a medical training initiative. We interviewed a rich variety of stakeholders and analyzed the data from the perspective of the readiness for change framework, which resulted in more clarity about the factors that influenced the “wall of goodwill” that we met during the implementation. One of our other strengths is that we used the readiness for change theory as a lens to conduct and analyze the interviews. Some might interpret the use of a theoretical framework as a lens as introducing a bias, which limits the validity of the findings.

A limitation is the small size of the group of participants in the study and the fact that the MedGezel program was executed in just one country. It would be most interesting to learn from implementation experiences in a variety of countries with different contexts and cultures. Moreover, future research must address in more detail how to cope with the medical habitus when it comes to education and pedagogical improvements for future health. Several participants spoke about management issues that we considered to be relatively easy to deal with. How could one recognize convenience arguments and distinguish them from issues that are more important? There is still a lot to learn about change management in the specific context of medical education.

### Recommendations

- Learn from difficulties and obstacles during the implementation of a medical educational innovation and publish the resulting insights;
- Follow the advice presented in theories of change. In our experience, we did not sufficiently take into account the useful knowledge offered by theories of change, and therefore several program implementations within our scope were not well planned [19];
- Spend energy on professional cultural issues if open culture is a pillar to build on or if hierarchy is at stake;
- Align with corporate strategy and be prepared to encounter hurdles if the workforce has different routines than the desired ones;
- Persist. Obstacles are only detours in the right direction and not a reason to quit. Learn from these obstacles and redirect your course based on a better plan.

## Conclusion

We introduced a novel form of clinical empathy education and believed that the generally positive reactions promised that it would be successfully implemented. However, despite the work of an energetic coordinator, the program hit a ‘wall of goodwill’ and ultimately experienced implementation obstacles and difficulties. Investigating the causes of the resistance revealed an internal struggle of stakeholders who embraced the vision but did not follow through or act accordingly in practice. At the level of the organization, there was a lack of strong leadership and there were some management hurdles, but these issues did not seem to be fatal for the implementation of the program. Rather, the most impactful explanation was found from a professional cultural perspective, which revealed an established medical culture with unwritten rules and rituals clashing with a new training initiative which gave clerks a new role with more status and the opportunity to critically observe the performance of their superiors. Despite these hurdles, we shall persist in our implementation efforts, only now paying greater attention to the supervisors. Medical culture must be addressed in any future endeavor, which may require, for instance, framing participating physicians as courageous change agents for the benefit of future health care.

## Data Availability

The datasets generated and/or analyzed during the current study are not publicly available due to the personal nature of the interviews with the participants and the agreement that the interviews would not be retraceable to the person, but the datasets are available from the corresponding author on reasonable request.
